# Novel contributor to chemotherapy resistance: an interferon-dependent subtype of cancer-associated fibroblast

**DOI:** 10.1186/s43556-023-00123-5

**Published:** 2023-04-20

**Authors:** Chu Xiao, Chunxiang Li, Jie He

**Affiliations:** Department of Thoracic Surgery, National Cancer Center/National Clinical Research Center for Cancer/Cancer Hospital, Chinese Academy of Medical Sciences and Peking Union Medical College, Beijing, 100021 China

In a recent article published in Cancer Cell, Ma et al. [1] identified a novel cancer-associated fibroblast (CAF) subtype termed interferon-regulated CAF (irCAF) in bladder cancer (BC), which was specifically induced by the type I interferon (IFN) within the tumor microenvironment (TME) and closely associated with unfavorable prognosis and low therapy response in patients.

CAF is a ubiquitous cellular component within solid tumor tissues and predominantly shows cancer-protectable abilities during tumorigenesis compared to their relatively less reported cancer-inhibiting effects [2]. Previous studies have profiled the multiple cancer-promoting effects of CAFs in the TME, such as secreting extracellular matrix proteins, inducing inflammation and angiogenesis, as well as engaging in the development of the immunosuppressive microenvironment, and these functions in part result in chemotherapy or immunotherapy resistance along with facilitating tumor progression [3]. Intriguingly, different solid tumor types have similar CAF populations, so it is of great significance to further explore whether CAFs can be targeted therapeutically in cancer. So far, the scarce CAF-specific markers and the less understanding of CAFs’community heterogeneity displayed across the course of cancer development are critical obstacles to developing CAF-targeted strategies [3].

To characterize the CAF population in bladder cancer (BC), Ma et al. performed single-cell RNA sequencing (scRNA-seq) on tumor specimens and matching para-cancerous tissues from naïve therapy patients (*n* = 8) and identified four CAF clusters: the general CAF subtype myofibroblastic CAFs (mCAFs), inflammatory CAFs (iCAFs), resting fibroblasts, and a novel subtype irCAFs characterized by SLC14A1 unique expression (Fig. 1). In-situ immunofluorescence staining showed that irCAFs were distributed within tumor tissues rather than para-cancerous tissues, while they highly expressed growth factors including NRG1, BMP5, STC1, and WNT5A, suggesting the nonresting potency of irCAFs in the TME. Furthermore, the authors validated the prevalence of the irCAF subtype in BCs by reanalyzing public datasets, and SLC14A1 coupled with COL1A1 (canonical fibroblast marker) could serve as the distinguishable marker of irCAFs.

**Fig. 1:**
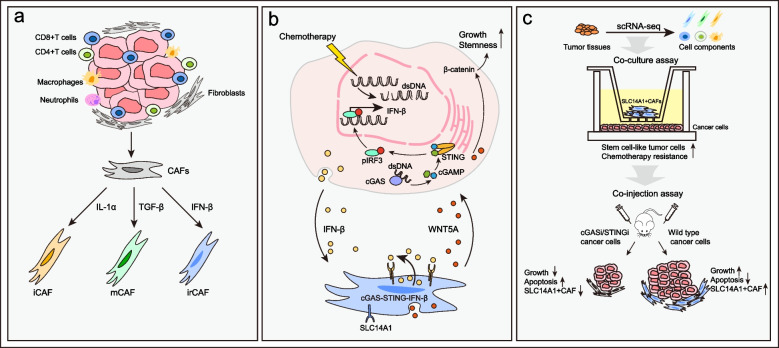
Mechanisms for irCAF evolution and their cancer stemness-promoting function. **a** Different cytokines induce CAF differentiation into distinct subtypes. **b** The regulatory loop between cancer cells and irCAFs. Cancer cells secret IFN-β to promote irCAF formation, while irCAFs generate WNT5A to enhance cancer stemness. **c** The brief exploration schematic diagram of the work. irCAFs are identified in bladder cancer tissues by scRNA-seq. The ex vivo and in vivo experiments validate the cancer-promoting effects of irCAFs

Critically, Ma et al. found that the number of irCAFs was markedly correlated with patients’ poor prognosis and less response to neoadjuvant chemotherapy or immunotherapy. To address the underpinning mechanisms by which irCAFs are linked to cancer progression, authors performed transcriptome sequencing of BC cell lines following co-culture with irCAFs, and the result showed that the co-culture system increased the fraction of stem cell-like cancer cells and activated cancer stem cell signatures. Consistent with the survival analysis of patient cohorts, cancer growth and chemotherapy nonresponsiveness were augmented after cancer cells co-cultured with irCAFs ex vivo or co-subcutaneous injected with irCAFs into mice models. Ma and colleagues checked the impacts of the upregulated growth factors in irCAFs, and WNT5A was identified as the primary contributor to the cancer stemness-promoting effects of irCAFs on cancer cells. As the downstream stimulus receiver, the cancer cell-intrinsic β-catenin pathway mediated the stem marker expression and chemotherapy resistance.

Ma et al. found that irCAFs could not maintain their viability in the regular ex vivo culture condition. In contrast, irCAF proportion was increased and maintained when co-culturing with cancer cells, similarly, the addition of supernatant of cancer cells into the culture medium also attenuated irCAF loss in vitro. This finding suggests the critical effects of tumor secretions on CAF survival and subcluster formation in TME. Studies have shown that the differentiation of specific CAF subtypes can be induced by different cytokines within TME, such as IL-1α for iCAFs or TGF-β for mCAFs [3]. In line with the prominent activation of the type I IFN response signaling in irCAFs, the expression and nuclear translocation of STAT1 was enhanced in irCAFs, and IFN-β treatment indeed increased the SLC14A1^+^irCAF fractions among bulk CAFs. Ma et al. further validated the transcription factor-dependent regulatory effects of STAT1 on SLC14A1 and WNT5A by chromatin immunoprecipitation assay, and STAT1 knockdown in CAFs promoted chemotherapy sensitivity observed in the mice model. Collectively, Ma et al. proved the underlying regulatory function of type I IFN on CAF differentiation in TME.

The critical step of innate immunity activation initiated by aberrant nucleic acid generation in cells includes the activated downstream nucleic acid sensing adaptors promoting type I IFN expression. Ma et al. found that the activation of cancer endogenous cyclic GMP-AMP synthase (cGAS)-STING signaling pathway was the primary initiator of IFN-β production in TME. In such a context, chemotherapy treatment, which could trigger cancer DNA double-strand breaks, activated the cGAS-STING pathway and subsequently induced more SLC14A1^+^CAF formation. This is likely a novel understanding of therapeutic resistance mediated by CAFs. Especially Ma et al. identified that phosphorylated STING is only correlated with poor survival when accompanied by high COL1A1, which suggested that irCAF induction was likely a key contributor to the unfavorable response of patients to STING agonists in clinical. In parallel, either STAT1 knockdown in CAFs or cGAS/STING knockout in cancer cells significantly repressed tumor growth and SLC14A1^+^CAF formation in vivo, concomitantly enhancing the chemotherapy sensitivity.

Taken together, Ma and colleagues identify a novel cancer-promoting CAF subtype in bladder cancer and reveal that the cancer-derived IFN-β is the core inducer for the subtype formation. Notably, their work proposes a previously unknown cGAS-STING-IFN-dependent CAF remodeling course, which especially contributes to chemotherapy resistance in BC. This study again highlights the plasticity and heterogeneity of CAFs accompanied by cancer progression, and the discovery of irCAF will be meaningful to help to find novel combination therapy regimens that may bolster the efficacy of chemotherapy. In addition, the cGAS-STING pathway has been profoundly studied along with several developed agonists that display favorable efficacy in clinical trials [4]. The work of Ma et al. suggests that inducible irCAF is likely to be a factor for poor response to these drugs. Combination therapies comprising targeting CAFs, cancer immunity, and cytotoxic drugs are likely promising therapeutic regimens in the future.

There are still some critical issues that need further elucidation. For example, how common is irCAF formation and maintenance in other cancer types, and whether feasible targets aiming at irCAF deletion can be implemented? Except for interacting with cancer cells, CAFs have been found to affect the functions of other non-malignant cells in TME to modulate the niche for tumor growth and therapy responses [5]. And the high proportion of immunotherapy-unresponsive patients who have activated irCAF phenotypes according to Ma’s study may suggest the potential roles of irCAFs in immunotherapy inefficiency. Therefore, given the unaccounted interplay between the newly classified irCAFs and other immune cells within TME, more explorations are needed to clarify the overall effects and dominance of irCAF in cancer advances and therapies.

## Data Availability

Not applicable.
